# Cytoplasmic Compartmentalization of the Fetal piRNA Pathway in Mice

**DOI:** 10.1371/journal.pgen.1000764

**Published:** 2009-12-11

**Authors:** Alexei A. Aravin, Godfried W. van der Heijden, Julio Castañeda, Vasily V. Vagin, Gregory J. Hannon, Alex Bortvin

**Affiliations:** 1 Watson School of Biological Sciences, Howard Hughes Medical Institute, Cold Spring Harbor Laboratory, Cold Spring Harbor, New York, United States of America; 2 Department of Embryology, Carnegie Institution of Washington, Baltimore, Maryland, United States of America; Stanford University School of Medicine, United States of America

## Abstract

Derepression of transposable elements (TEs) in the course of epigenetic reprogramming of the mouse embryonic germline necessitates the existence of a robust defense that is comprised of PIWI/piRNA pathway and *de novo* DNA methylation machinery. To gain further insight into biogenesis and function of piRNAs, we studied the intracellular localization of piRNA pathway components and used the combination of genetic, molecular, and cell biological approaches to examine the performance of the piRNA pathway in germ cells of mice lacking Maelstrom (MAEL), an evolutionarily conserved protein implicated in transposon silencing in fruit flies and mice. Here we show that principal components of the fetal piRNA pathway, MILI and MIWI2 proteins, localize to two distinct types of germinal cytoplasmic granules and exhibit differential association with components of the mRNA degradation/translational repression machinery. The first type of granules, pi-bodies, contains the MILI-TDRD1 module of the piRNA pathway and is likely equivalent to the enigmatic “cementing material” first described in electron micrographs of rat gonocytes over 35 years ago. The second type of granules, piP-bodies, harbors the MIWI2-TDRD9-MAEL module of the piRNA pathway and signature components of P-bodies, GW182, DCP1a, DDX6/p54, and XRN1 proteins. piP-bodies are found predominantly in the proximity of pi-bodies and the two frequently share mouse VASA homolog (MVH) protein, an RNA helicase. In *Mael*-mutant gonocytes, MIWI2, TDRD9, and MVH are lost from piP-bodies, whereas no effects on pi-body composition are observed. Further analysis revealed that MAEL appears to specifically facilitate MIWI2-dependent aspects of the piRNA pathway including biogenesis of secondary piRNAs, *de novo* DNA methylation, and efficient downregulation of TEs. Cumulatively, our data reveal elaborate cytoplasmic compartmentalization of the fetal piRNA pathway that relies on MAEL function.

## Author Summary

Vast territories of animal genomes are populated by numerous types of mobile genetic elements (or transposons) that act predominantly as selfish parasites unconcerned with the impact of their activity on the well-being of the host. In response to the danger posed by transposons, organisms have evolved a defensive mechanism that employs a particular class of small RNAs known as piRNAs to identify and selectively silence transposons. We have studied the subcellular organization of such a defensive mechanism, the piRNA pathway, in germ cells of mouse male embryos. We discovered that key proteins involved in the genesis of small RNAs, MILI and MIWI2, occupy specific domains within the cytoplasm of germ cells. Surprisingly, MIWI2 shares its domain with proteins known to degrade RNAs and repress synthesis of cellular proteins, thus raising a possibility of cooperation of the two mechanisms in transposon defense. Genetic ablation of MAEL, a protein also found within the MIWI2 domain, disrupts normal MIWI2 localization and piRNA production leading to transposon activation. This study demonstrates that an elaborate compartmentalization of the defensive mechanism is required for the efficient recognition and destruction of active transposons in germ cells of mice.

## Introduction

Small RNAs play crucial roles in the control of many aspects of cell growth and differentiation. An ancient class of small RNAs, known as piRNAs for their association with PIWI proteins, specializes in the protection of genomes from the adverse effects of transposable elements (TEs) [1],[2]. The defensive role of piRNAs is most prominent in germ cells whose genomic integrity is key for propagation. Genetic and molecular studies of PIWI proteins and piRNAs have began to unravel genome defensive mechanisms in *Drosophila* [3]–[5], *C. elegans* [6],[7], zebrafish [8],[9] and mice [10]–[13], where piRNAs are implicated in transposon silencing at both the transcriptional and post-transcriptional levels.

The mouse genome encodes three PIWI-like proteins, MIWI (or PIWIL1, Mouse Genomic Information), MILI (MGI: PIWIL2) and MIWI2 (MGI: PIWIL4), all of which play essential and non-redundant roles in spermatogenesis [12]–[16]. MIWI is expressed after birth in pachytene spermatocytes and spermatids [14]. *Miwi*-null spermatocytes arrest post-meiotically at the round spermatid stage [14]. Although the basis of this developmental defect is unknown, MIWI has been posited to act in translational control. MILI and MIWI2 are the only PIWI-family proteins required for transposon silencing in fetal gonocytes [10],[13]. MILI is first detected in germ cells at E12.5 and persists in adult testes where it is expressed during spermatogenesis until the round spermatid stage. MIWI2 expression is restricted to gonocytes and coincides with *de novo* DNA methylation of TEs (E15.5 - P2) [10]. MILI is present exclusively in the cytoplasm in numerous perinuclear granules, while MIWI2 is most abundant in gonocyte nuclei but also appears in prominent cytoplasmic granules, which are exclusive of, though often adjacent to, those that contain MILI.

MILI and MIWI2 play distinct but complementary roles in silencing transposons in developing male germ cells, and this is reflected in their interaction with discrete populations of small RNAs [10]. MILI binds 26 nt piRNAs that are predominantly derived from sense strands of TE transcripts, while MIWI2 shows a preference for 28 nt piRNAs derived from anti-sense TE transcripts. Together, these RNAs show features of the ping-pong amplification cycle that both allows honing and adaptation of the system and consumes transposon transcripts during the generation of new small RNAs.

Originally described in *Drosophila*, the ping-pong cycle is constituted of two classes of piRNAs [3],[17]. In flies, primary piRNAs are derived from specialized generative loci, piRNA clusters, and these mainly direct Aubergine complexes to recognize and cleave transposon mRNAs. Cleavage of the transposon message generates a secondary piRNA, which is sense-oriented with respect to the element. This small RNA complexes with AGO3, which uses the secondary piRNA as a guide to cleave antisense transposon RNAs, presumably derived from piRNA clusters, thus forming a complete cycle. In mice, MILI and MIWI2 form a similar cycle with transposon mRNAs and piRNA clusters; however, the origins of primary and secondary piRNAs appears to be reversed [10]. Transposon-derived, sense species appear to be the primary piRNAs, while cluster-derived RNAs remain antisense but in this system are formed as secondary species.

Many studies have supported critical roles for DNA methylation in transposon silencing [18],[19]. This highlights the significance of the dynamic reprogramming of methylation patterns that occur in developing germ cells [20]. Starting at E11.5, mouse embryonic germ cells of both sexes lose DNA methylation patterns inherited from parental gametes on imprinted genes and transposons. Sexually dimorphic differentiation can be observed at E12.5. Oocytes enter meiosis at E13.5 while male germ cells, or gonocytes, undergo mitotic arrest at E14.5 - 15.5 and remain quiescent until P2. The *de novo* DNA methylation of transposons is established in these non-cycling gonocytes. Previous studies have suggested that a catalytically inactive member of the DNA methyltransferase family, DNMT3L, acts upstream of the active, *de novo* methyltransferases, DNMT3A and DNMT3B, to determine methylation patterns [18], [21]–[24]. Genetic and molecular characterization of interactions between methyltransferases and the piRNA pathway are consistent with PIWI complexes directing DNMT3L, and indirectly active methyltransferases, to target loci based upon their bound small RNA guides [10].

While a general picture of the piRNA-based defensive mechanism in the mammalian germline has begun to emerge, much remains to be discovered about this pathway, the mechanisms which enable its selective recognition of mobile genetic elements, and the routes used to selectively silence transposons. Previous studies have shown that mutations in fruit fly and mouse *Maelstrom* (*Mael*) genes derepress transposons [25],[26]. MAEL is a protein of unknown biochemical function with a non-canonical HMG-box and a unique domain homologous to the DnaQ-H 3′-5′ exonuclease [27]. Importantly, *Mael*-mutant mice share germ cell phenotypes with mutants lacking *Mili*, *Miwi2* and *Dnmt3l* [26]. These include a complete block of spermatogenesis due to apoptosis during meiotic prophase I, defects in homologous chromosome synapsis, DNA damage, reduced DNA methylation, and derepression of L1 retrotransposons. To gain further insights in transposon silencing, we examined the subcellular organization and function of the piRNA pathway in mouse fetal gonocytes lacking MAEL.

## Results

### MAEL co-localizes with MIWI2 in cytoplasmic granules of fetal gonocytes

**Figure 1:**
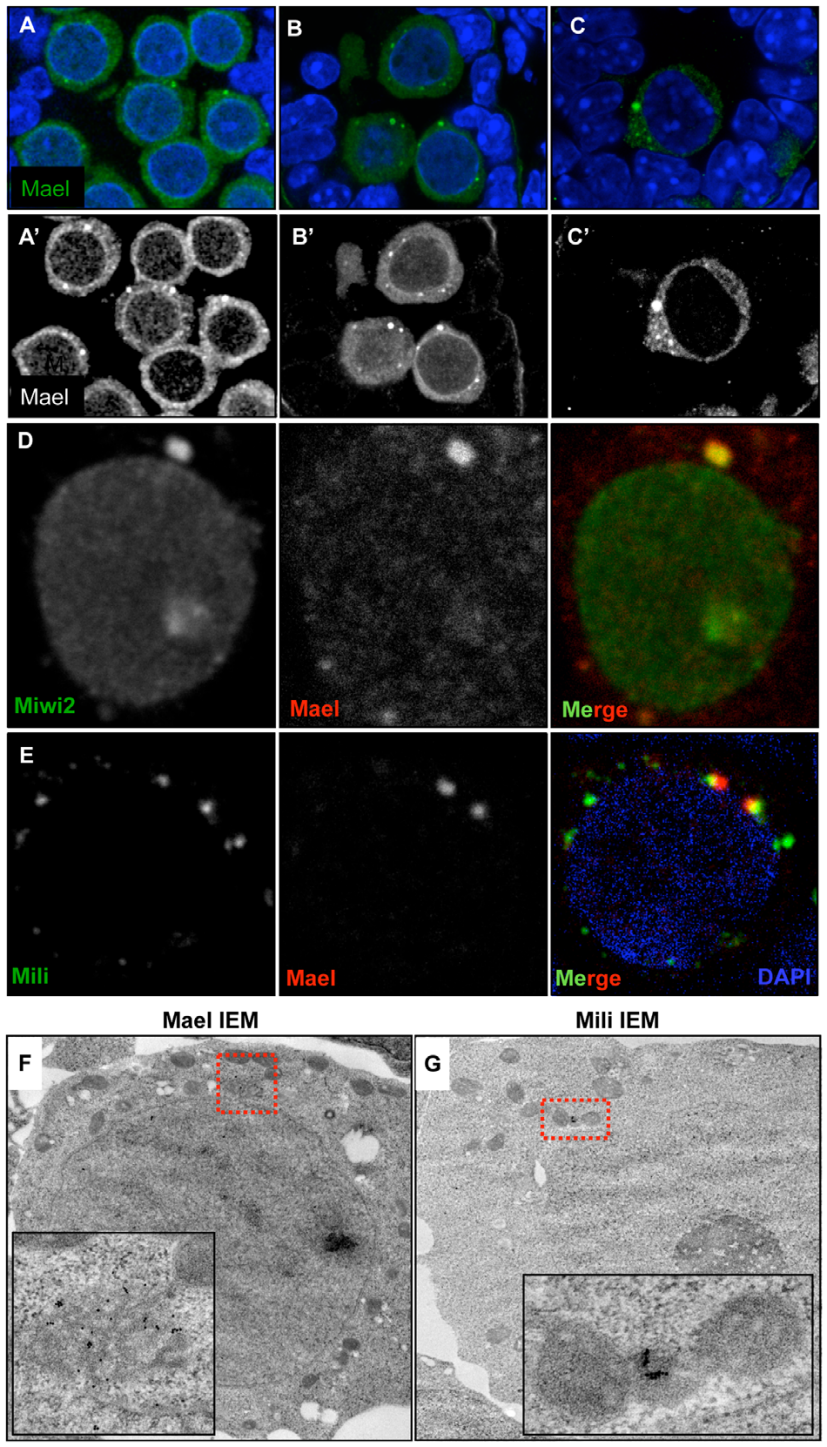
Localization of MAEL, MIWI2, and MILI to cytoplasmic granules in gonocytes. (A–C’) Sections of E16.5 (A, A’), E18.5 (B, B’) and P2 (C, C’) testes stained with anti-MAEL antibody (green) and DAPI (blue). (A’–C’) anti-MAEL signal in black/white to emphasize nuclear signal. (D) Localization of MIWI2 and MAEL in a E18.5 gonocyte. MAEL was detected by a directly labeled antibody, resulting in a diminished detection level. (E) Localization of MILI and MAEL in a E18.5 gonocyte. MAEL was detected with a directly labeled antibody, resulting in a diminished detection level. (F–G) Localization of MAEL (F) and MILI (G) in E18.5 testes by immuno-electron microscopy. Localization of MAEL shows it to be a component of electron dense perinuclear granules. Immuno-gold labeling for MILI identified the granules observed by IF to be intermitochondrial cement (ICM). The inset shows a blow-up of the region indicated in the red box.

To begin to address the function of MAEL in the piRNA pathway, we used a highly specific anti-MAEL antibody [26] to study the dynamics of its localization during the developmental window (E14.5 - P2) that encompasses the period of *de novo* DNA methylation of TEs in the mouse male germline. We examined MAEL localization in gonocytes of E14.5, E16.5, E18.5 and P2 wild-type testes. MAEL was initially found throughout the cytoplasm at E14.5 (data not shown), but by E16.5 it started to accumulate in up to 10 prominent perinuclear granules per gonocyte (Figure 1A). In addition, MAEL was present in gonocyte nuclei at modest levels at E16.5 and E18.5 but not at P2 (Figure 1A’–1C’). This pattern of MAEL localization is reminiscent of its localization in germ cells of adult males where MAEL is a prominent component of cytoplasmic germinal granules (nuage) in spermatocytes and the chromatoid body in round spermatids [26]. Cytoplasm-nucleus shuttling of MAEL was previously observed in *Drosophila* oogenesis and further supported by the immuno-electron microscopy (Immuno-EM) in mouse spermatocytes [28].

Interestingly, MAEL localization resembled that of MIWI2 [10] raising a possibility of co-localization of the two proteins. Indeed, double immunostaining revealed that MIWI2 and MAEL were present in same cytoplasmic granules from E16.5 through P2 (98% co-localization among the total of 385 granules scored in 68 examined gonocytes). In addition, both proteins were distributed homogeneously in gonocyte nuclei where MIWI2 accumulated at significantly higher levels compared to MAEL (Figure 1D). In contrast, MILI and MAEL failed to co-localize (Figure 1E). However, consistent with a prior report, MILI and MIWI2/MAEL granules were often found in close proximity (93%, N = 406, n = 79) (Figure 1E). Since MILI granules were also more abundant, only a minority of them associated with MAEL/MIWI2 granules.

These results revealed a dynamic pattern of MAEL localization in gonocytes during the period of *de novo* DNA methylation of TEs that is consistent with a role in the PIWI/piRNA pathway. In particular, these observations suggested a closer functional relationship of MAEL with MIWI2 than with MILI.

### MAEL/MIWI2 and MILI localize to distinct nuages

The granular appearance of MILI, MIWI2 and MAEL by antibody staining raised the intriguing possibility that these proteins might localize to the enigmatic nuage structures of fetal gonocytes. Nuages, electron-dense perinuclear material, have been recognized in the germ cells of at least 80 species throughout the animal kingdom [29],[30]. Accumulating evidence points to a role for nuages in RNA metabolism and storage [31]–[33].

To identify the MIWI2/MAEL and MILI nuages, we used immuno-EM. When we used an anti-MAEL antibody, we found gold particles labeling characteristic perinuclear, membrane-free organelles of 0.5 to 1.5 µm in diameter (Figure 1F). These structures were reminiscent of chromatoid bodies in that they contained sponge-like networks of moderately electron-dense material with numerous pores in between. As was the case for MAEL localization within the chromatoid bodies of round spermatids, MAEL-targeted gold particles were most often associated with the dense material of the organelle. In contrast, MILI-targeted gold particles were predominantly found to localize to smaller electron-dense patches positioned between adjacent mitochondria (Figure 1G). These areas appeared similar to the intermitochondrial cement (IMC) observed over 35 years ago in rat gonocytes [29]. These results show that MAEL/MIWI2 and MILI localize to different types of nuages suggesting their functional specialization in piRNA biogenesis and RNA metabolism.

### Additional germ cell–specific components of germinal granules

**Figure 2:**
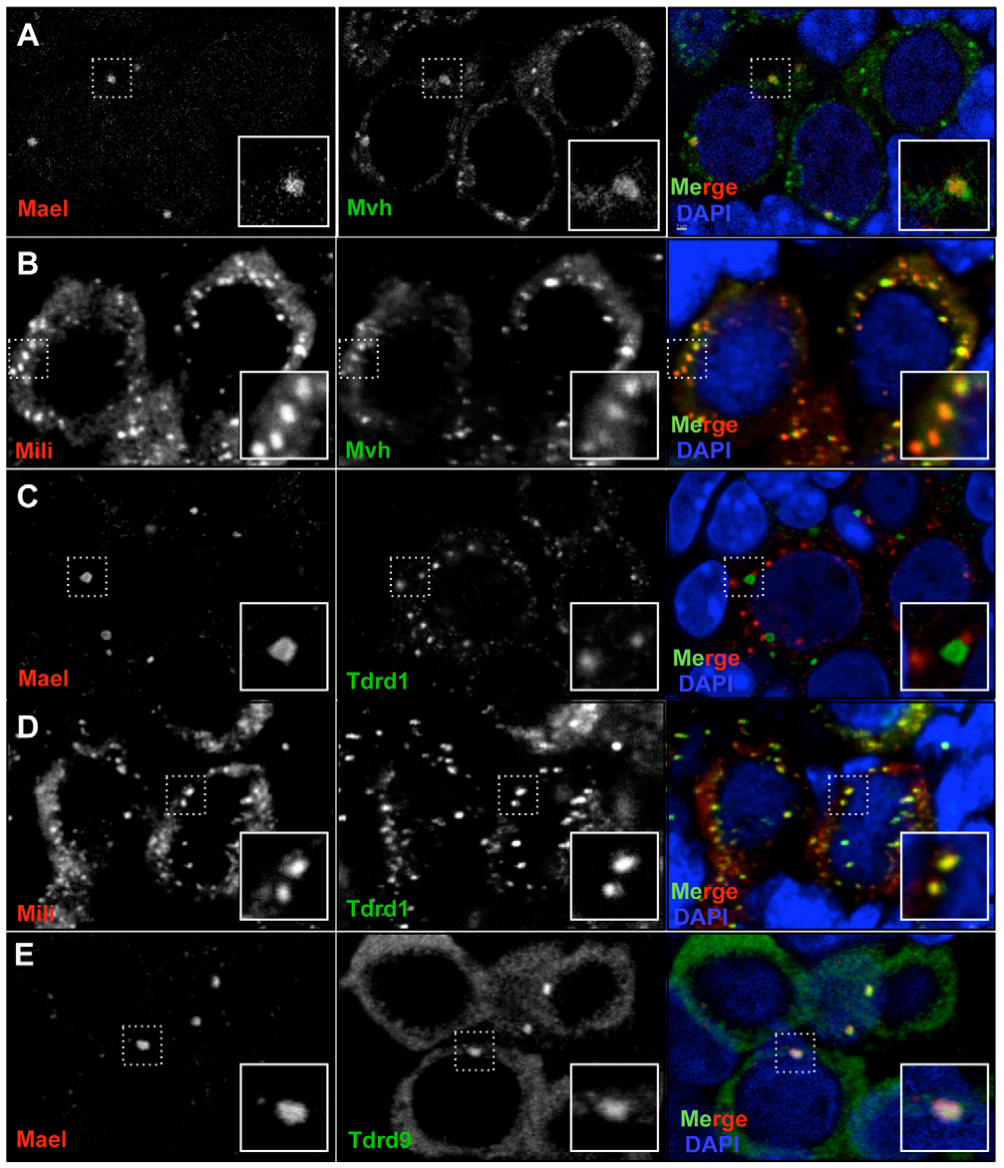
Localization of germ cell specific proteins to granules. (A–E) Localization of germ cell specific proteins MVH (A, B), TDRD1 (C, D) and TDRD9 (E) combined with MAEL (A, C, E) or MILI (B, D). DNA is labeled with DAPI (blue). (A) MAEL and MVH co-localize in larger granules. MVH additionally localizes to smaller granules. (B) Double labeling with MILI and MVH reveals the latter to be a component of IMC. (C) TDRD1 is a known component of IMC. It localizes to numerous smaller granules that are frequently adjacent to MAEL granules. (D) MILI and TDRD1 co-localize to IMC. (E) TDRD9 co-localizes with MAEL in granules.

To aid in our understanding of the nature and function of MILI and MIWI2-MAEL granules, we examined the location of other proteins implicated in RNA metabolism or that have been shown to have granular localization patterns in germ cells. The germ-cell specific RNA helicase, VASA, is a known component of nuage and pole plasm in *Drosophila* and has recently been shown to be functional in the piRNA pathway [34]. Mouse VASA homolog (MVH, MGI: DDX4) is required for IMC formation and spermatogenesis [35],[36]. By probing E18.5 gonocytes with anti-MVH antibody, we found that MVH localizes to cytoplasmic granules of different sizes (Figure 2A and 2B). Double-labeling with anti-MAEL (Figure 2A) or anti-MILI (Figure 2B) antibodies identified MVH as a component of both MIWI2-MAEL- and MILI-positive granules. However, MVH was found in MAEL granules at a slightly lower frequency compared to MIWI2 (85% co-localization and 15% adjacent, N = 135, n = 60). These results demonstrate that VASA, a universal germ cell specific marker across the animal kingdom, localizes to both types of nuage in mouse fetal gonocytes thus underscoring their related functions in RNA metabolism.

MVH is required for the correct localization of Tudor repeat domain-containing protein 1 (TDRD1) [37], which is required for efficient piRNA production [38],[39]. TDRD1 has been reported to localize to IMC and is essential for its formation [40]. Consistent with that, we did not find overlap between TDRD1- and MAEL-containing granules of E18.5 gonocytes (Figure 2C) whereas a complete co-localization of MILI and TDRD1 was observed (Figure 2D). These results corroborate our IEM result that identifies MILI as a component of IMC.

Another mouse Tudor domain protein, TDRD9, is a homolog of Spindle-E that is essential for VASA and MAEL localization, and production of piRNAs in *Drosophila* [28],[34]. Recently, we found this Tudor domain protein to specifically immunopurify with MIWI2 and not MILI [39]. Consistent with this data, in E18.5 gonocytes, we observed that TDRD9 resides exclusively in MIWI2-MAEL granules but is absent from MILI-TDRD1 granules (Figure 2E). Considered together, the results of our localization studies revealed elaborate spatial compartmentalization of two modules of the ping-pong cycle of piRNA biogenesis, MILI-TDRD1 and MIWI2-TDRD9-MAEL, into distinct cytoplasmic bodies thus implying their functional specialization in metabolism of transposon mRNAs.

### The MIWI2/MAEL granule is a modified P-body

Previously, we have reported that MAEL localizes to the chromatoid body in round spermatids [26]. This structure contains a large number of proteins including MVH, TDRD1, TDRD6, TDRD7 and TDRD9, MILI, and MIWI [31]. Although the precise function of the chromatoid body is not clear, it is now generally recognized that it serves as an RNA processing center [32],[33],[41]. This is, among others, indicated by the presence of components of Processing bodies (P-bodies). P-bodies are structures in which non-translating mRNA can be either degraded or stored [42],[43]. Since the chromatoid body and MILI, and MIWI2 granules share several components we asked if P-body components are also present in these organelles.

**Figure 3:**
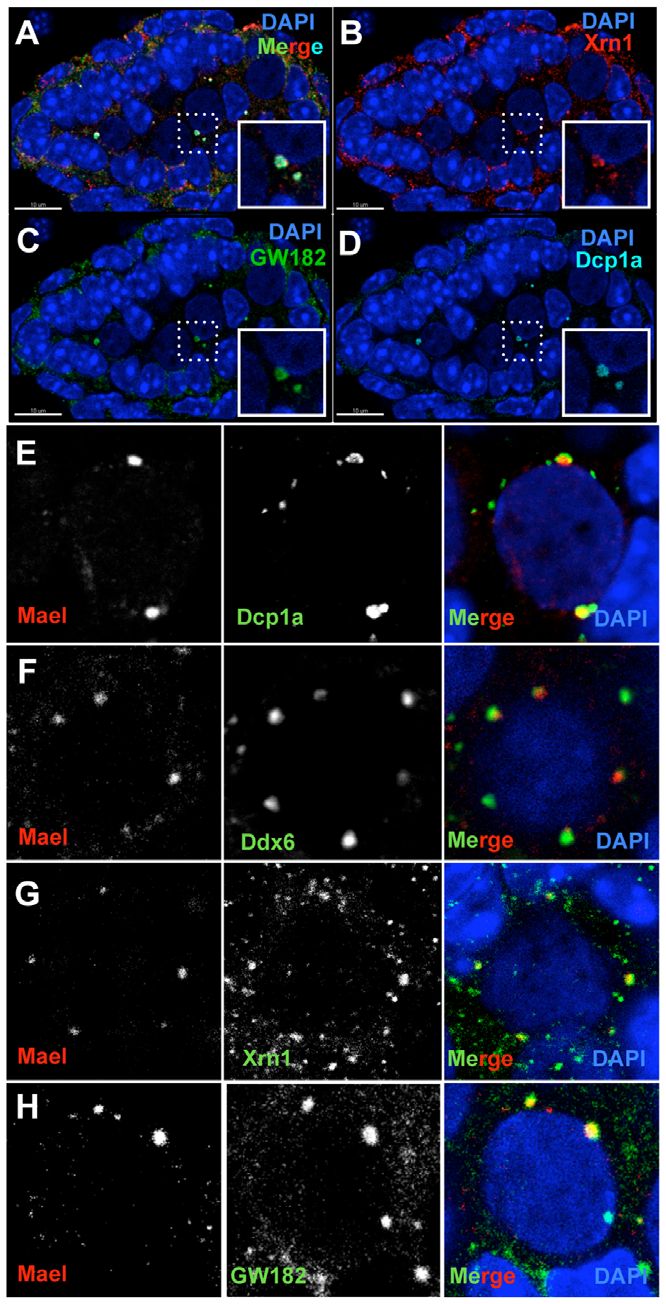
The MIWI2/MAEL granule is a modified P-body. (A–D) Localization of P-bodies in gonocytes. Cross section seminiferous tubule from wild-type testis (E18.5) were probed with antibodies against XRN1 (B), GW182 (C) and DCP1a (D). (E–H) Co-localization of MAEL and P-body components DCP1a (E), DDX6 (F), XRN1 (G), and GW182 (H) in piP-bodies.

We focused on four canonical P-body proteins: RNA helicase DDX6/p54, DCP1a, a subunit of decapping enzyme, XRN1 5′ to 3′ exonuclease and GW182 (MGI: TNRC6a), a component of the canonical RNAi-induced silencing complex [44]–[46]. Simultaneous detection of DCP1a, XRN1 and GW182 in gonocytes showed that, just as in somatic cells, these factors co-localized (99%, N = 230) (Figure 3A–3D).

The overall appearance and size of P-bodies was very similar to MIWI2/MAEL granules suggesting that these might be one and the same. Labeling experiments with MAEL indeed identified these four P-body proteins as components of the MIWI2/MAEL granule (Figure 3E–3H). To determine if all P-bodies contained MAEL we performed double staining for MAEL and DCP1a. The majority of MAEL granules (95%, N = 503, n = 60) contained DCP1a, and by extension DDX6, XRN1 and GW182. However, a substantial portion of P-bodies identified by DCP1a labeling did not contain MAEL (39%, N = 774, n = 60). These results revealed that the canonical RNA degradation machinery and the piRNA pathway exhibit significant cytoplasmic co-localization in the fetal germline.

Furthermore, closer examination of co-localization of MAEL with P-body proteins revealed that MAEL and MIWI2 occupy the core of the granule while GW182 was found in the surrounding shell (Figure 3 and Figure S1). Similarly to GW182, DCP1a was also observed to coat the MAEL/MIWI2 core (Figure 3E). In contrast, 5′ to 3′ exonuclease XRN1 was found in the core of the granule (Figure 3G).

Overall, our studies demonstrate that nuages are major cytoplasmic sites of accumulation of piRNA pathway proteins and serve to spatially and functionally compartmentalize two modules of the ping-pong cycle of piRNA biogenesis. Based on our data, we proposed to name MILI-TDRD1 nuages “pi-bodies” and MIWI2-TDRD9-MAEL nuages “piP-bodies” to indicate simultaneous presence of piRNA pathway and P-body components in the latter structure.

### Loss of MAEL results in ultrastructural changes of piP-bodies

**Figure 4:**
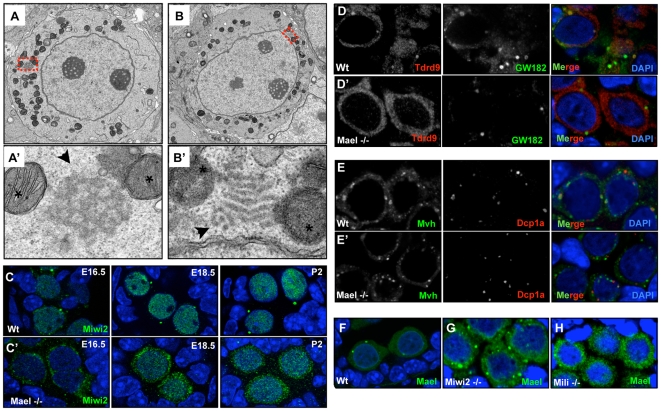
Altered structure of piP-bodies in the absence of MAEL. (A–B’) EM images of piP-bodies in E18.5 wild-type (A, A’) and *Mael*-mutant gonocytes (B, B’). Magnified regions (A’, B’) are indicated in red in (A,B). *Mael*-deficient gonocytes exhibit change in overall appearance of the MIWI2/MAEL granule (indicated with black arrow, compare (A’, B’)). Association with mitochondria (indicated with asterisks in (D’, E’)) was not affected. (C–E’) The effect of *Mael* deficiency on (C–C’) MIWI2, (D–D’) TDRD9 and (E–E’) MVH localization in gonocytes. In wild-type testes, MIWI2 localizes to cytoplasmic granules and becomes nuclear at E16.5 (C, left panel). Later (E18.5, middle panel and P2, right panel), MIWI2 localization in the nucleus becomes more pronounced. (C’) In absence of MAEL, MIWI2 does not localize to cytoplasmic granules and nuclear localization is delayed (E16.5, left panel, E18.5, middle panel and P2 right panel). (D–D’) In wild-type gonocytes TDRD9 co-localizes with GW182 in piP bodies. No TDRD9 accumulations are observed in the *Mael* mutant. (E) In wild-type gonocytes, MVH and DCP1a frequently co-localize in large granules. (E’) In absence of MAEL this association is lost. (F–H) MAEL localization in wild-type (F), *Miwi2*-deficient (G), and *Mili*-deficient (H) testes. In *Miwi2*-mutant animals, MAEL is still recruited to piP-bodies but cytoplasmic levels are significantly higher than in wild-type gonocytes. MAEL localization in *Mili*-mutant animals is largely disrupted. Virtually no MAEL granules are observed and no nuclear localization is detected.

To address the role of MAEL in piP-body structure and function, we examined wild-type and *Mael*-mutant gonocytes at the ultrastructural level. In EM images of wild-type E18.5 gonocytes, we could readily identify piP-bodies based upon their sponge-like appearance, rounded shape, size, and proximity to mitochondria (Figure 4A and 4A’, and Figure S2). Similarly, we were able to identify piP-bodies in *Mael*-mutant gonocytes, however, their morphology was visibly changed (Figure 4B and 4B’, and Figure S2). Specifically, piP-bodies had lost their sponge-like appearance, and instead of a network of electron-dense, fuzzy canals, they appeared as parallel stacks of thick electron-dense smooth barrels that lacked obvious connections with each other. Despite these changes, this organelle has retained features characteristic of piP-bodies that distinguished it from P-bodies observed in somatic cells [47],[48]. Thus, lack of MAEL has a dramatic effect on ultrastructural organization of the piP-body but does not seem to completely prevent accumulation and/or assembly of some of its components.

### MAEL is required for localization of germ cell factors to P-bodies

The overall change in piP-body morphology prompted us to examine which of its components depend on MAEL for their normal localization. By probing gonocytes with corresponding antibodies we observed no effect of the *mael* mutation on the localization of the P-body components XRN1, DCP1a, DDX6 and GW182 (Figure S3). These proteins co-localized with each other at frequencies similar to those observed in wild-type gonocytes. Hence, MAEL is not required for P-body formation in germ cells. However, we did notice the absence of very large piP-bodies (as in Figure 3A), possibly indicating the loss of other components.

Indeed, *mael* mutation had a profound effect on MIWI2 localization (Figure 4C and 4C’). First and foremost, MIWI2 failed to accumulate in the piP-bodies and instead was distributed more or less evenly throughout the cytoplasm of mutant cells. Second, nuclear accumulation of MIWI2 was delayed, with the protein becoming detectable in the nucleus at E18.5 rather than at E16.5 (compare left panels in Figure 4C and 4C’). The effect of the *mael* mutation extended to TDRD9 as this protein completely failed to accumulate in cytoplasmic granules (Figure 4D).

To address the question whether loss of MAEL affected protein composition of pi-bodies, we examined the localization of MILI and TDRD1. We did not see any effect on their appearance (Figure S4 and data not shown). Likewise, MVH localization did not seem to change dramatically in the absence of MAEL since MVH granules persisted in the cytoplasm (Figure 4E and 4E’). However, because MVH was found to be present in both piP-bodies and pi-bodies in wild-type gonocytes, we performed MVH and DCP1a double-labeling and quantified the number of DCP1a granules that 1) co-localized with MVH, 2) associated with MVH granules or 3) were free from MVH in wild-type and *Mael*-mutant E18.5 gonocytes (for visual representation see Figure S5). We observed a sharp reduction in MVH-DCP1a labeled piP-bodies in *Mael*-mutant gonocytes [39% in the wt (N = 660) vs. 3% (N = 974) in the *mael* KO, Figure 4E’]. *Mael*-deficient gonocytes also show a small increase in DCP1a and MVH associated granules (40% in wt vs. 48% in the *mael* KO) and a major increase in solitary DCP1a granules (21% in the wt vs. 49% in the *mael* KO). These observations suggest that loss of MAEL did not affect MVH localization to pi-bodies but had blocked its entry into piP-bodies.

Cumulatively, these results suggest that MAEL plays essential role in the formation piP-bodies by facilitating MIWI2, TDRD9, and MVH localization to these granules. In contrast, pi-body formation is unaffected by the lack of MAEL and piP-bodies.

### MILI is required for correct localization of MAEL

To assess whether the dependency of MIWI2 on MAEL was symmetrical we determined the localization of MAEL in *Miwi2*-mutant mice. Disruption of *Miwi2* had only a minor effect on MAEL, apparent as a decrease in the intensity of the signal of piP-bodies and an increase in uniform staining in the cytoplasm (Figure 4G). Previously, a requirement for MILI for MIWI2 localization to cytoplasmic granules has been shown [10]. The asymmetrical dependency for localization of MIWI2 and MAEL prompted us to determine the localization of MAEL in absence of MILI. A complete loss of MAEL accumulation to piP-bodies and nuclei was observed in *Mili*-mutant gonocytes (Figure 4H). These results suggest that molecular processes in pi-bodies are essential for piP-body formation.

### piRNA production is perturbed in the absence of MAEL

**Figure 5:**
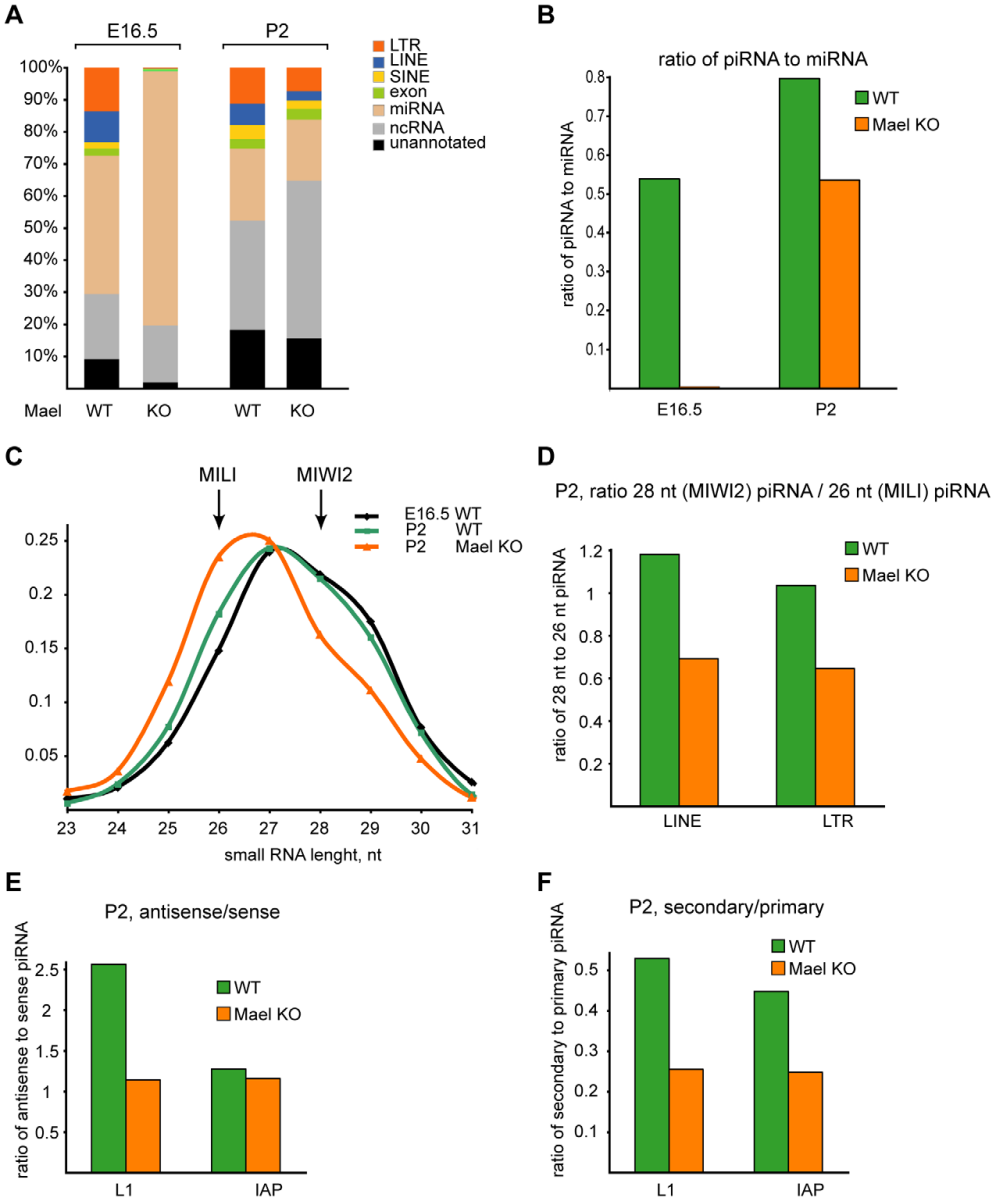
The effect of *Mael* deficiency on piRNA expression in gonocytes. (A) Annotation of small RNAs cloned from testes of wild-type and *Mael*-mutant animals. The right panel shows the ratio of LINE and LTR piRNA to miRNA. (B) The size range of piRNAs in the *mael* mutant. Total cellular piRNA populations are composed of two complexes, MILI, with average piRNA length 26 nt. and MIWI2 with average piRNA length 28 nt. The length profile reflects the ratio of both complexes in the cell. In wild-type animals the ratio of does not significantly change between E16.5 and P2. MIWI2-associated piRNAs (28 nt) corresponding to both LINE and LTR retrotransposons are significantly reduced in *Mael*-mutant animals. (C) The ratio of sense to antisense piRNAs for L1 and IAP retrotransposons. The amount of LINE L1 antisense piRNA is reduced in *Mael*-deficient animals at P2. In contrast, amount of LTR IAP antisense piRNA remains stable. (D) The ratio of secondary to primary piRNAs for L1 and IAP retrotransposons. The amount of secondary piRNAs that correspond to LINE L1 and LTR IAP is reduced in *Mael*-deficient animals at P2.

To examine directly the performance of the piRNA pathway in *Mael*-mutant gonocytes, we performed deep sequencing of 19–33 nt small RNAs, a size range that includes both miRNAs (20–22nt) and piRNAs (25–30nt) (Figure 5 and Table S1). We examined two time points - E16.5 corresponding to the period of robust *de novo* DNA methylation and P2 that immediately follows completion of TE silencing. This analysis revealed that miRNAs appeared unaffected by the *mael* mutation at both examined time points, permitting normalization of small RNA libraries derived from different samples. In contrast to miRNAs, we observed a dramatic difference in piRNA production between wild-type and *Mael*-mutant E16.5 testes (Figure 5A). Firstly, unlike in the wild-type, sequencing of the *Mael* mutant revealed virtual absence of piRNAs (see Table S1 for details). When normalized to miRNAs, transposon-derived small RNAs were 100-fold less abundant in the *Mael* mutant than in the wild type. Secondly, transposon-derived small RNA in *Mael* mutant lacked characteristic sequence features of fetal piRNAs – preferential 5′ uridine (1U), a signature of primary piRNA processing, and adenine at position 10 (10A), a signature of secondary piRNA processing by a ping-pong mechanism [10]. For example, in contrast to wild type L1 piRNAs 72.5% of which had 5′ uridine, only 36% of small RNAs started with 1U in the *Mael* mutant. Likewise, the 10A fraction decreased from 46% in the wild-type piRNAs to 24% in the transposon-derived small RNA in *Mael*-mutant testes suggesting a complete lack of amplification by the ping-pong mechanism. Finally, the size distribution of these small RNAs sequences lacked the peak at 25–30 nt characteristic of genuine piRNAs but was evenly distributed across all size ranges (Figure S6). This analysis suggested that piRNA production has not commenced in the absence of functional MAEL in E16.5 gonocytes. This finding agrees with the results of protein localization studies at E16.5 that showed absence of MIWI2 and TDRD9 in piP-bodies and in the nucleus.

Surprisingly, despite the severe defect in piRNA biogenesis at E16.5, piRNA populations had largely recovered in the P2 *Mael* mutant yielding only ∼1.5-fold differences between *Mael* mutant and wild-type samples (Figure 5A and Table S1). To gain insight into the mechanisms by which loss of MAEL impacts piRNA production, we examined sequence features of small RNA populations in *Mael* mutant animals at P2. piRNAs bound to MILI and MIIW2 have characteristic sizes, being respectively 26 and 28 nucleotides long. This holds true for piRNAs from both E16.5 and P2 wild-type samples (Figure 5B). The relative abundance of these two species is altered in the *Mael* mutant at P2 due to specific reduction of 28 nt piRNAs. Thus, despite the overall recovery of piRNA levels in P2 gonocytes, MIWI2-interacting piRNAs were underrepresented in the *Mael* mutant.

MIWI2 is preferentially associated with antisense piRNAs, which are enriched for secondary species emerging from the ping-pong cycle. In the *Mael* mutant, the relative abundance of antisense piRNAs corresponding to L1 is reduced by 2.5 fold, while more modest effects are seen on IAP antisense/sense ratios (Figure 5C). For both IAP and L1, secondary piRNAs are depleted (Figure 5D). Considered together, these observations point to a preferential effect of the *mael* mutation on MIWI2 complexes and to a corresponding disruption of the efficient operation of the ping-pong cycle. This results in a delay in the accumulation of piRNA populations and a shift in their character overall.

### Gonocytes in the *Mael* mutant are not delayed in their cell cycle arrest

To eliminate the possibility that the impacts that we observed resulted from a developmental delay in *Mael*-mutant gonocytes, we examined the entry of these cells into the cell cycle arrest phase that characterizes the period of *de novo* DNA methylation. Heterozygote *mael* mice were mated and pregnant females injected twice with nucleoside analog 5-ethynyl-2′-deoxyuridine (EdU) to label replicating DNA in maternal and fetal tissues [49]. One group of animals was injected at 15 and 16 dpc (days post coitum), while the other was treated at 16 and 17 dpc (Figure S7). Wild-type and *Mael*-mutant gonocytes (identified by MVH staining) were indistinguishable in their arrest at both time points. Moreover, the localization of DNMT3L and DNMT3A2 exhibited similar patterns in wild-type and mutant E13.5–E16.5 gonocytes (Figure S8). We concluded that the effects of *mael* mutation on the piRNA pathway likely indicate a direct, functional relationship.

### *de novo* DNA methylation in *Mael*-mutant testes

Lesions of *Mili* or *Miwi2* lead to defects in the *de novo* DNA methylation of transposable elements. Mutation of *Mael* delayed the onset of piRNA production but at P2 levels were only ∼1.5 time less than in wild type. To determine whether this delay and partial loss affects *de novo* DNA methylation of L1 elements we determined their methylation status. To obtain pure gonocytes, we prepared testicular cell suspensions and sorted them manually after staining for MVH. Genomic DNA from wild-type and mutant gonocytes was treated with sodium bisulfite and used as a template for amplification of L1 fragments using primers designed to the 5′ regions of the element consensus sequences. Sequencing of individual amplicons (Table 1) and analysis by QUMA software [50] revealed reduced methylation of L1 at E16.5 but modification was substantially recovered by E18.5. In neonatal gonocytes of wild-type and *Mael*-mutant pups, DNA methylation levels of L1 elements were identical. Loss of MAEL, therefore, does not derail the acquisition of *de novo* DNA methylation of these transposable elements.

**Table 1:** Methylation status of 5′ region of L1 elements.

	E16.5	E18.5	P2
**L1, wild type**	61% (22)	81% (25)	82 *
**L1, mael−/−**	49% (15)	85% (13)	85% (23)

Shown are percentages of CpG dinucleotides that are methylated. In between brackets the number of reads is shown.

*data from [13].

### Disruption of *Mael* perturbs post-transcriptional transposon silencing in fetal gonocytes

**Figure 6:**
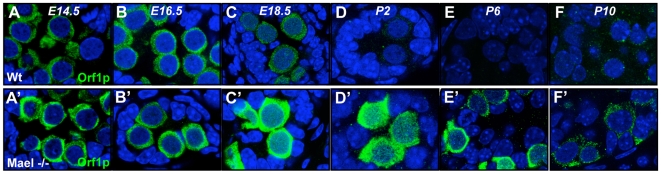
Release of post-transcriptional silencing of L1 elements in *Mael*-mutant gonocytes. (A–F’) Localization of L1 encoded ORF1p in testes of wild-type (A–F) and *Mael*-mutant (A’–F’) animals from E14.5 to P10. Overall levels in wild-type animals decreased after E16.5 whereas in *Mael*-mutant testis ORF1p levels increased at E14.5 onwards. From P2 on signal for ORF1p gradually decreased.

The protein composition of piP-bodies raised a possibility of perturbed post-transcriptional or translational silencing of transposons in the absence of MAEL. Along these lines, we examined the dynamics of expression of L1-encoded ORF1p protein in wild-type and *Mael*-mutant fetal and neonatal testes (E14.5 to P10) (Figure 6). Consistent with incomplete DNA methylation of L1 in E16.5 wild-type gonocytes (Table 1), ORF1p accumulation was most prominent at this stage (Figure 6B). By E18.5 cytoplasmic ORF1p levels began to decline reflecting on the 20% gain of DNA methylation of L1 in the genome (Figure 6C). Minor ORF1p signal was observed in P2 gonocytes and no protein was detect at P6 (Figure 6D and 6E). Consistent with our prior work [26], re-appearance ORF1p expression was observed at the onset of meiosis (P10) (Figure 6F). These results demonstrate that in wild-type gonocytes, L1 ORF1p levels closely follow and parallel the extent of DNA methylation of their genomic loci from E16.5 onward. In contrast to the wild type, *Mael*-mutant gonocytes persistently exhibited elevated but eventually gradually declining ORF1p levels throughout the examined period despite normal levels of DNA methylation of L1 from E18.5 on (Figure 6A’–6F’, Table 1). This result demonstrated that the loss of MAEL impacted not only the onset of production of piRNAs and DNA methylation of transposons but also had a significant effect on protein levels. We have reached a similar conclusion by examining the dynamics and levels of expression of IAP-encoded Gag protein (data not shown). Considered together, these data suggest that MAEL contributes to post-transcriptional silencing of transposons by means of its association with P-body components.

## Discussion

In the male germline of mice, efficient transposon silencing is achieved by a concerted action of the piRNA pathway and *de novo* DNA methylation machinery [10],[13]. To gain further insights into the cellular and molecular aspects of piRNA pathway function, we focused on MAEL, an evolutionarily conserved protein previously implicated in small RNA functions in fly and mouse germ cells. In the course of this study, we obtained evidence of elaborate spatial and functional compartmentalization of the piRNA pathway in fetal gonocytes, and of an important role of MAEL in this process.

We demonstrate that two principle components of the fetal piRNA pathway, MILI and MIWI2, and their specific partner proteins of the Tudor domain repeat family, reside in distinct types of germinal cytoplasmic granules. Based on the ultrastructural appearance, proximity to mitochondria and the presence of TDRD1 protein, we concluded that the MILI-containing granules, or pi-bodies, are likely equivalent to the “cementing material” between mitochondria first described in rodent gonocytes over 35 years ago. Less numerous piP-bodies contain the MIWI2-TDRD9-MAEL module of the piRNA pathway and four hallmark components of P-bodies, GW182, DCP1a, DDX6/p54 and XRN1. In addition to their unique constituents, pi- and piP-bodies share MVH protein, an evolutionarily conserved marker of germ cells throughout the animal kingdom. Cumulatively, these observations establish that mouse fetal gonocytes possess two types of specialized cytoplasmic organelles that contain distinct modules of the piRNA pathway and exhibit differential association with the mRNA degradation/translational repression machinery.

The piP-body is a striking component of the elaborate cytoplasmic compartmentalization of the piRNA pathway. What logic underlies the formation of such a joint cytoplasmic body that combines mRNA degradation/translational machinery and the piRNA pathway in the gonocyte? P-body formation is believed to be the product of aggregation of translationally repressed mRNAs and associated proteins. Our data, however, suggest that the association of the piRNA machinery with P-bodies is not simple lumping of piRNA-protein complexes and P-body proteins together. First, MIWI2 and not MILI is found in piP-bodies. Therefore, there must be a mechanism that differentiates between the two proteins. Second, incorporation of the MIWI2-TDRD9 module and MVH into piP-bodies is not spontaneous but depends on MAEL function. Third, even following the incorporation into the piP-body, MIWI2 does not appear to form aggregates or stable complexes with P-body proteins as none of these were identified in our recent proteomic analysis of MIWI2 [39]. Finally, the ultrastructural appearance of the piP-body in MAEL-deficient gonocytes is clearly different from that of P-bodies and suggests presence of other component(s) in the piP-body independent of MAEL, MIWI2, TDRD9 and MVH. Together, these observations suggest the existence of a coordinated interplay of the MIWI2-TDRD9-MAEL module of the piRNA pathway with mRNA degradation/translational repression machinery. MAEL appears to play a crucial (direct or indirect) role in regulating this process.

Our data demonstrate that MAEL ensures robust defensive response to transposon derepression during DNA methylation reprogramming of the male germline. In the *Mael* mutant, MIWI2 fails to translocate to the nucleus by E16.5. This coincides with lack of piRNAs and lagging *de novo* DNA methylation in E16.5 gonocytes. However, eventual accumulation of MIWI2 in the nucleus parallels significant recovery of piRNA levels and completion of *de novo* DNA methylation during the E18.5 - P2 window. These results suggest that MAEL functions to facilitate MIWI2-dependent steps of the piRNA pathway. In contrast to the recovery of its nuclear localization, MIWI2 completely fails to localize to piP-bodies in the absence of MAEL. This defect in piP-body formation is accompanied by a prolonged accumulation of L1 ORF1p in *mael* postnatal prospermatogonia even after the completion of *de novo* DNA methylation. This result suggests that MAEL and the piP-body play an important role in efficient downregulation of transposon expression.

Our results further refine the relationship between MAEL with MILI and MIWI2. Consistent with our prior results, MILI is absolutely required for normal localization of MIWI2 and MAEL. Interestingly, lack of MIWI2 has only minor effect on MAEL localization and instead it is MAEL that is required to ensure normal MIWI2 localization and function. From this perspective, MAEL occupies an intermediate position between PIWI proteins in the fetal piRNA pathway.

Considering our data as a whole, our study reveals elaborate cytoplasmic compartmentalization of the piRNA pathway during the critical developmental window of DNA methylation of transposable elements in mouse fetal gonocytes. Future studies will be needed to further elucidate mechanistic relationships between PIWIs and their partner proteins TDRDs, MVH, MAEL and a recently described GASZ [51] in biogenesis and function of piRNAs.

## Materials and Methods

### Animals

The *Miwi2* and *Mili* knock-out strains are described in [12] and [15], respectively. 3xMyc-MIWI2 transgenic animals were described previously [10]. The *Mael* knock-out strain was described in [26].

### Immunofluorescence detection of protein localization

Immunofluorescence detection of protein localization on paraffin-embedded testicular sections was performed for *Mili* and *Miwi2* KO animals and respective controls as described previously [10]. For MIWI2 co-localization with MAEL, DDX6, TDRD1 and MVH, 3xMyc-MIWI2 transgenic animals and the same protocol were used. For *Mael* KO and control animals as well as other co-localization studies, frozen sections and the protocols described below were used.

Testis were fixed in freshly prepared 4% PFA for 2–3 hours at 4°C. After fixation, samples were washed in PBS and placed in 30% sucrose overnight at 4°C. Tissues were imbedded in OCT blocks and stored at −80°C. Sections were cut at 8 µm thickness. Testicular sections were washed for 15 minutes in PBS 0.05% Triton-X-100 and incubated for 1 hour at 37°C with blocking solution (PBS 0.05% Triton-X-100, 10% NGS, 3% BSA). Antibodies were applied (diluted in blocking solution) and incubated at room temperature overnight. The next day, slides were washed in PBS plus 0.05% Triton-X-100 for 5 minutes, followed by a 10 minute wash in PBS. Primary antibody labeling was followed by incubating for 2 hours at 37°C with the corresponding secondary antibody. After incubation, slides were washed in PBS and counterstained with DAPI. Vectashield (Vector) was used as anti-fading mounting solution. The Zenon kit (Invitrogen; Z-25306) was used for direct labeling of antibodies in case multiple rabbit derived antibodies were used. For imaging, a laser-scanning confocal microscope (SP2 or SP5; Leica, Exton, PA or Zeiss) was used.

### Immunoelectron microscopy

Testes were fixed at 4°C overnight in 4% paraformaldehyde; 0.2% picric acid; 2% Sucrose in 0.1M PBS, washed in PBS (3×10 min), dehydrated with ethanol, embedded into LR Gold and polymerized with UV at 4°C (2 days) and room temperature (2 days). Ultra thin sections were cut, collected on parlodion coated grids, stained with anti-MAEL [26], MILI antibody (Abcam, ab36764) or normal rabbit serum and 10 nm gold particle-conjugated secondary antibodies (Aurion), counterstained with uranyl acetate and visualized with an FEI Tecnai 12 electron microscope.

### Small RNA libraries

Small RNA isolation, library construction and annotation were performed as described previously [11].

### Bisulfite sequencing

Cell suspensions of wild-type or knockout embryos testis were made by incubating them in 500 ml GBSS (supplier) complemented with 5 mg trypsin (Worthington) for 10 minutes at 37C. Afterward, the tissue was carefully resuspended with P200 tips and filtered through a 40 µm filter (BD Falcon). An equal volume of serum was added as well as 150 ml 16% PFA (EMS). Cells were fixed for 15 minutes at 37C while gently shaking, after which they were spun down (1500 rpm, table centrifuge) for 10 minutes. Cells were resuspended in PBS (containing 4% FCS, 0.3% saponin and 0.05% sodium azide) and stored overnight at 4°C. Cells suspensions were stained with anti-MVH antibody and DAPI and around 300 gonocytes were manually selected with an epi-fluorescence-equipped blastocyst injection microscope setup. DNA was converted by using the EZ DNA methylation kit (D5020). For PCR amplification of pan-Line-1gf and pan-IAP sequences, primers as described in [13]. Product of the first PCR was purified by using QIAquick PCR purification kit (Qiagen). The second PCR was run on a gel and the band of corresponding size was collected. DNA was obtained by spinning down in GenElute spin columns (Sigma). PCR fragments were cloned into pGEM-T easy plasmids (Promega), transfected into Top10 cells (Invitrogen) and plated. Individual colonies were picked and sequenced.

### EdU labeling

Pregnant mice were injected with 150 µg of EdU dissolved in 300 µl saline at either day 15.5 and 16.5 or 17.5 and 18.5 days after detection of a vaginal plug. Mice were sacked 2 hours after the last injection and embryonic gonads were collected and processed as described above. EdU detection was performed prior to antibody staining, as described in the manual (Invitrogen).

### Antibodies

ORF1p (S.L. Martin; 1∶500), MILI (Abcam ab36764, 1∶750) and [11] (1∶200), MIWI2 ([10] 1∶750), MAEL [10], 1∶1000), TDRD1 and TDRD9 (S. Chuma, 1∶1000), MVH (Abcam ab13840, 1∶1000), DDX6 (Bethyl Lab. A300-460A,1∶250), GW182 (E.K. Chan), XRN1 (Bethyl Lab. A300-443A, 1∶250), DCP1a (Abnova, 55802-M06, 1∶500), DNMT3L and DNMT3A2 (S. Tajima,1∶1000), myc (Upstate clone 4A6, 1∶300).

## Supporting Information

Figure S1 GW182 forms an outer shell of the piP-body.
(2.16 MB TIF)

Figure S2 Electron micrograph images of piP-bodies in wild-type and *Mael*-mutant gonocytes. Examples of piP-bodies in wild-type (A) and in *Mael*-mutant (B) gonocytes. Regions indicated in red boxes are shown magnified below the overview.
(5.89 MB TIF)

Figure S3 Co-localization of P-body components in the *Mael*-mutant gonocytes. In *Mael*-deficient gonocytes, XRN-1, GW182, and DCP1a co-localization remains intact.
(4.93 MB TIF)

Figure S4 MILI localization in *Mael*-mutant gonocytes. Loss of MAEL does not affect the localization of MILI.
(2.45 MB TIF)

Figure S5 Associations of MVH and DCP1a in wild-type and *Mael*-mutant gonocytes. In wild-type gonocytes, 3 types of MVH-DCP1a localizations were observed: 1) overlapping, 2) associated granules, or 3) solitary granules. In the *Mael* mutant, virtually only associated or solitary DCP1a granules were present.
(1.18 MB TIF)

Figure S6 Size distributions of LINE and SINE small RNAs from E16.5 *Mael*-mutant testes.
(0.46 MB TIF)

Figure S7 Analysis of gonocyte cell cycle arrest in wild-type and *Mael*-mutant animals by EdU labeling of replicating DNA. (A) Schematic outline of the experiment–timing of EdU injections in two groups of animals and their sacrifice. (B) Representative DAPI/EdU/MVH staining of processed gonadal tissues. No EdU labeling was observed in gonocytes. (C) Quantification of EdU labeling in gonocytes. No EdU positive wild-type or *Mael*-mutant gonocytes were observed.
(1.56 MB TIF)

Figure S8 DNMT3L and DNMT3A2 expression in E16.5 *Mael*-mutant gonocytes. At day E13.5, no DNMT3L and DNMT3A2 staining was observed in wild-type and *Mael*-mutant gonocytes (data not shown). At day E16.5, all gonocytes showed prominent nuclear staining.
(3.82 MB TIF)

Table S1 Results of small RNA sequencing.
(0.14 MB TIF)
