# Supplementary figures and images for: Cytoplasmic Compartmentalization of the Fetal piRNA Pathway in Mice
Source: PLoS Genet. 2009 Dec 11;5(12):e1000764. doi: 10.1371/journal.pgen.1000764 (PMC2785470; doi:10.1371/journal.pgen.1000764)

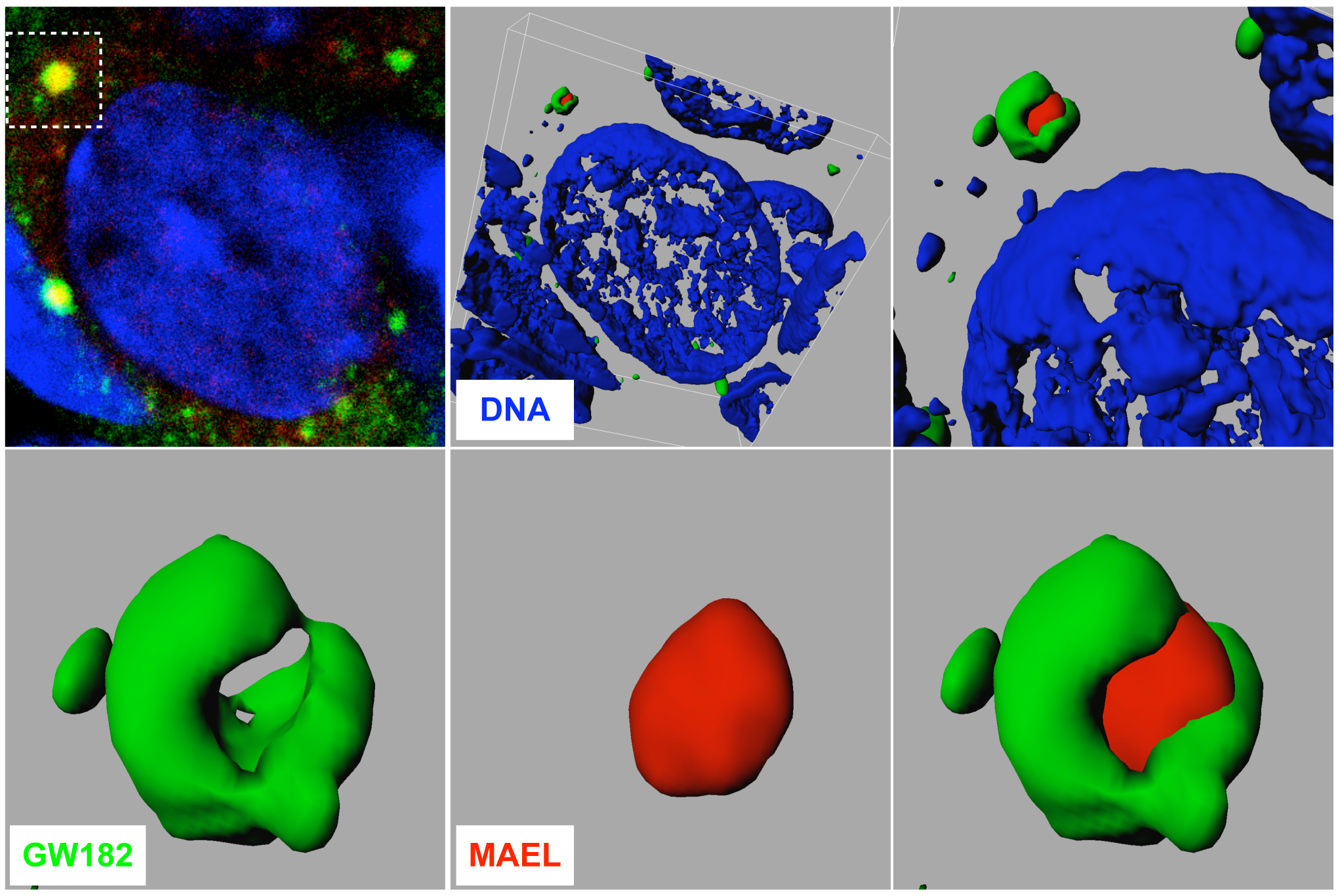

Supplement: Figure S1 — GW182 forms an outer shell of the piP-body. (2.16 MB TIF) [file pgen.1000764.s001.tif]

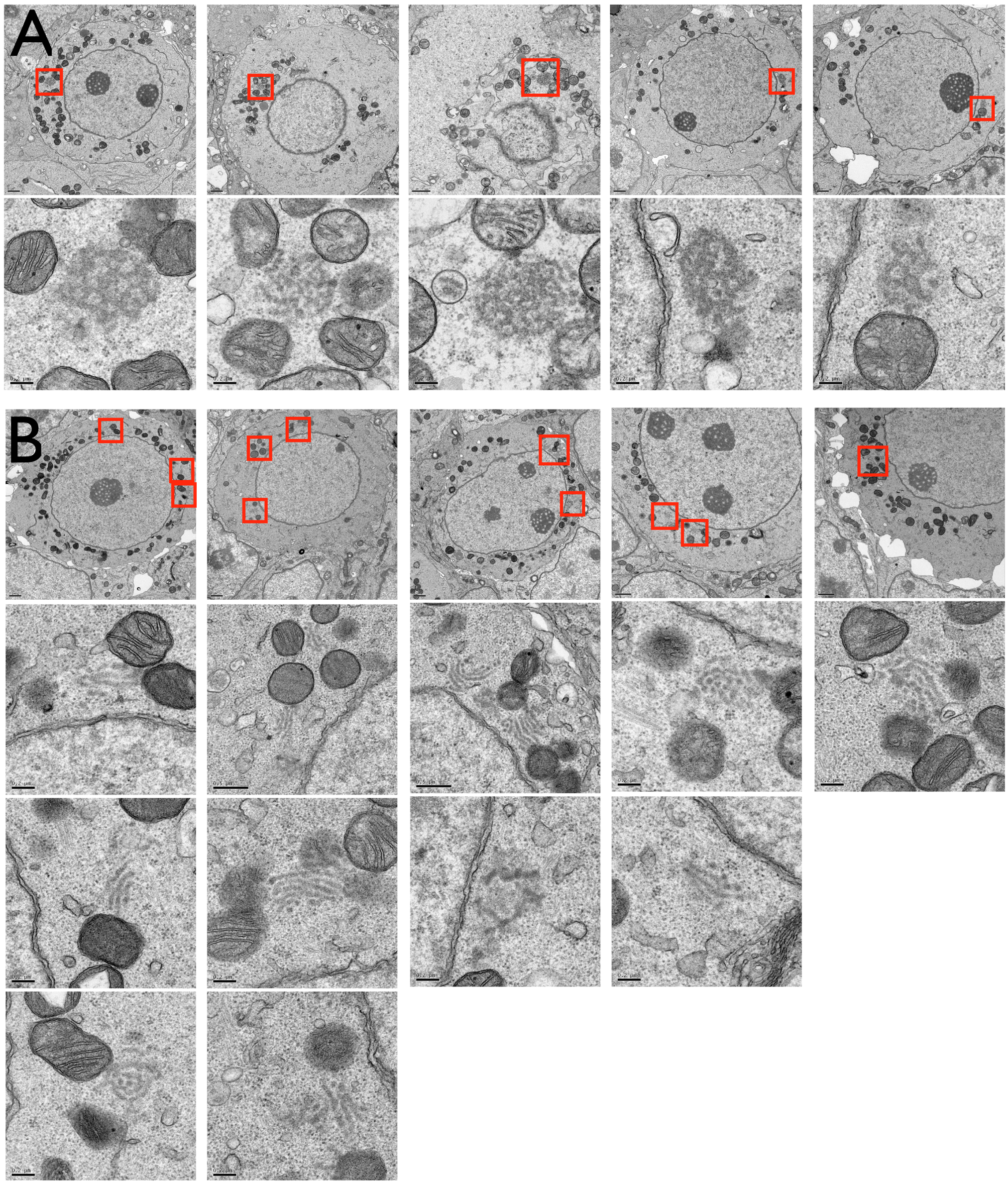

Supplement: Figure S2 — Electron micrograph images of piP-bodies in wild-type and Mael-mutant gonocytes. Examples of piP-bodies in wild-type (A) and in Mael-mutant (B) gonocytes. Regions indicated in red boxes are shown magnified below the overview. (5.89 MB TIF) [file pgen.1000764.s002.tif]

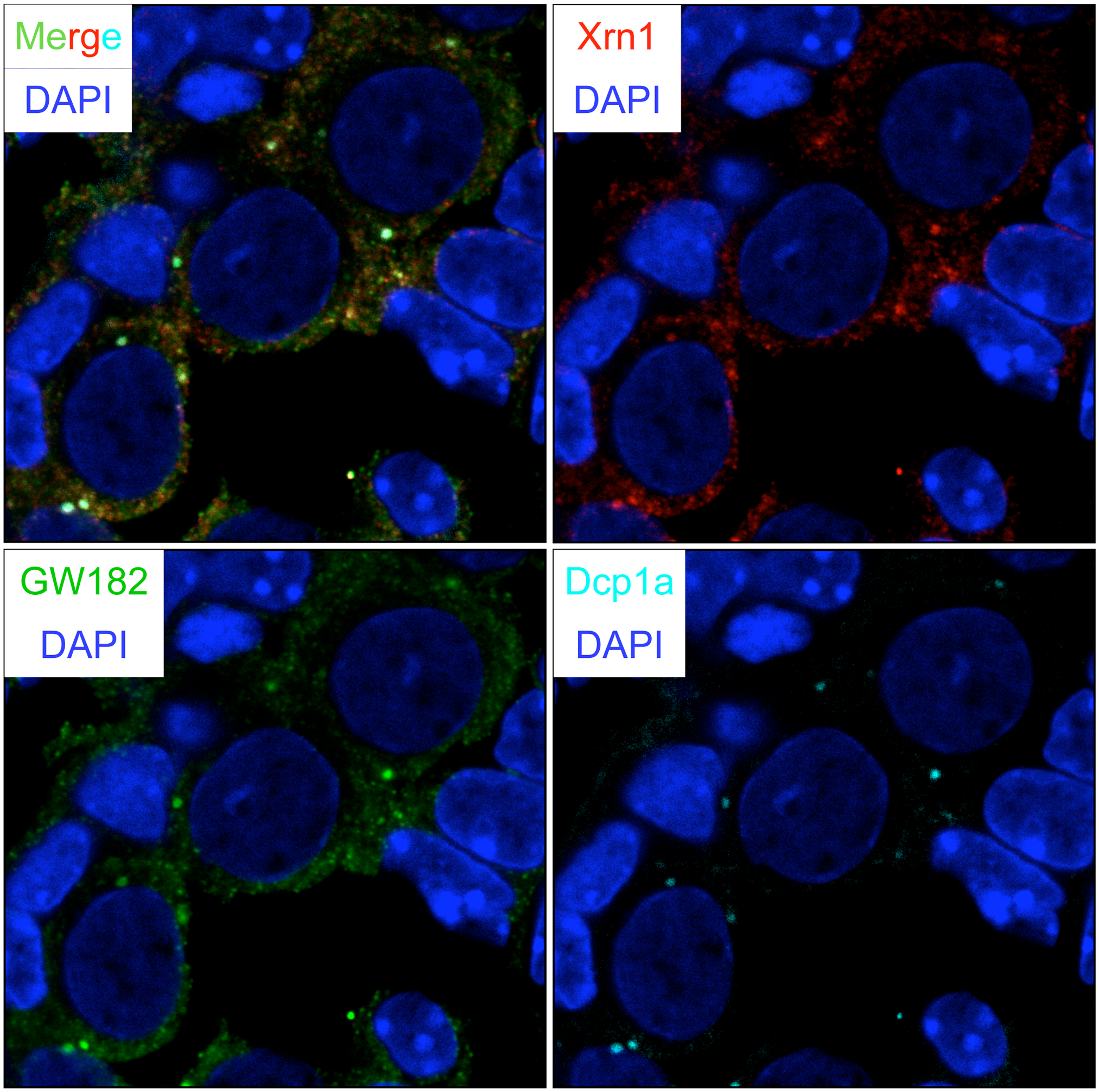

Supplement: Figure S3 — Co-localization of P-body components in the Mael-mutant gonocytes. In Mael-deficient gonocytes, XRN-1, GW182, and DCP1a co-localization remains intact. (4.93 MB TIF) [file pgen.1000764.s003.tif]

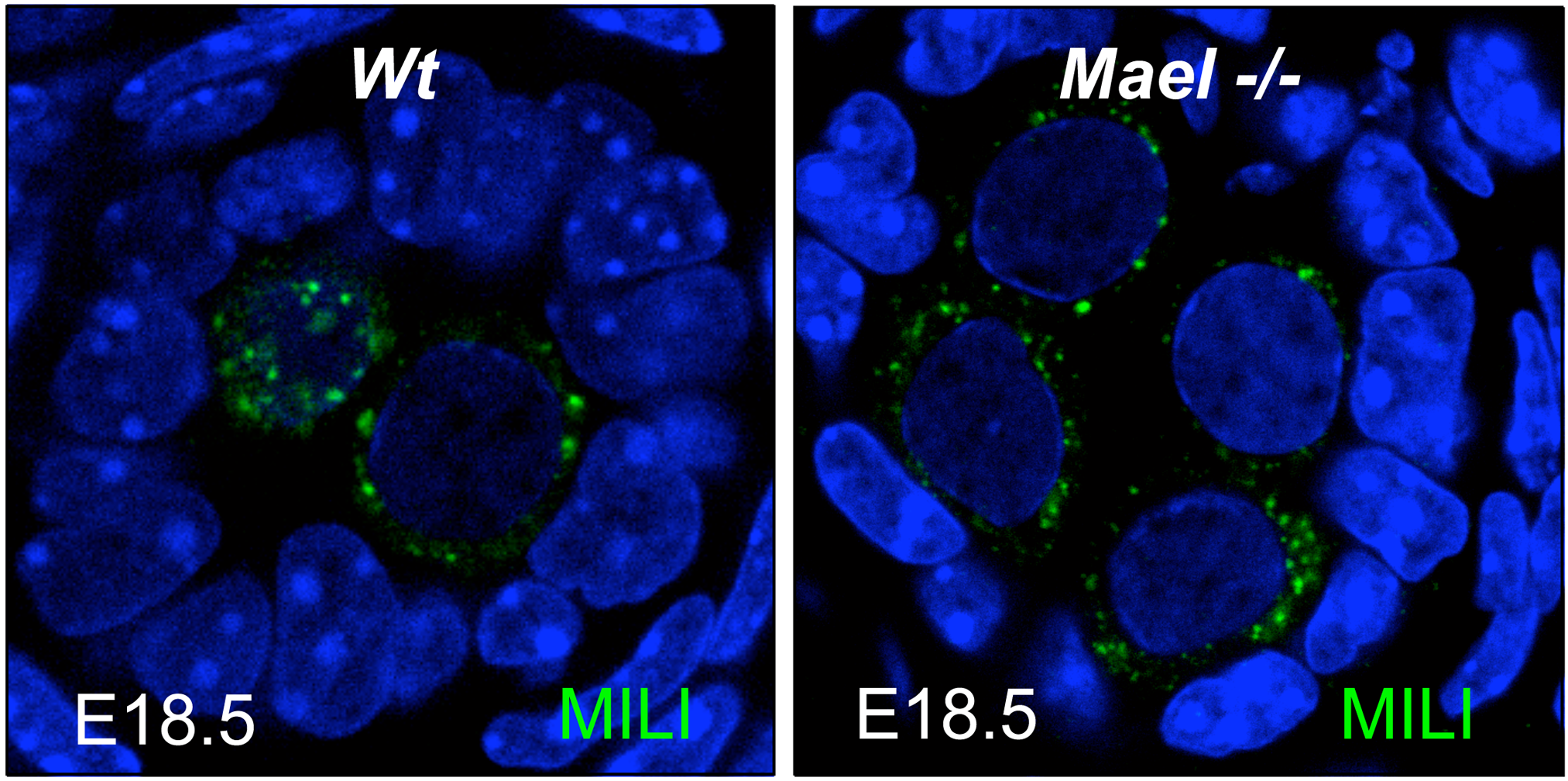

Supplement: Figure S4 — MILI localization in Mael-mutant gonocytes. Loss of MAEL does not affect the localization of MILI. (2.45 MB TIF) [file pgen.1000764.s004.tif]

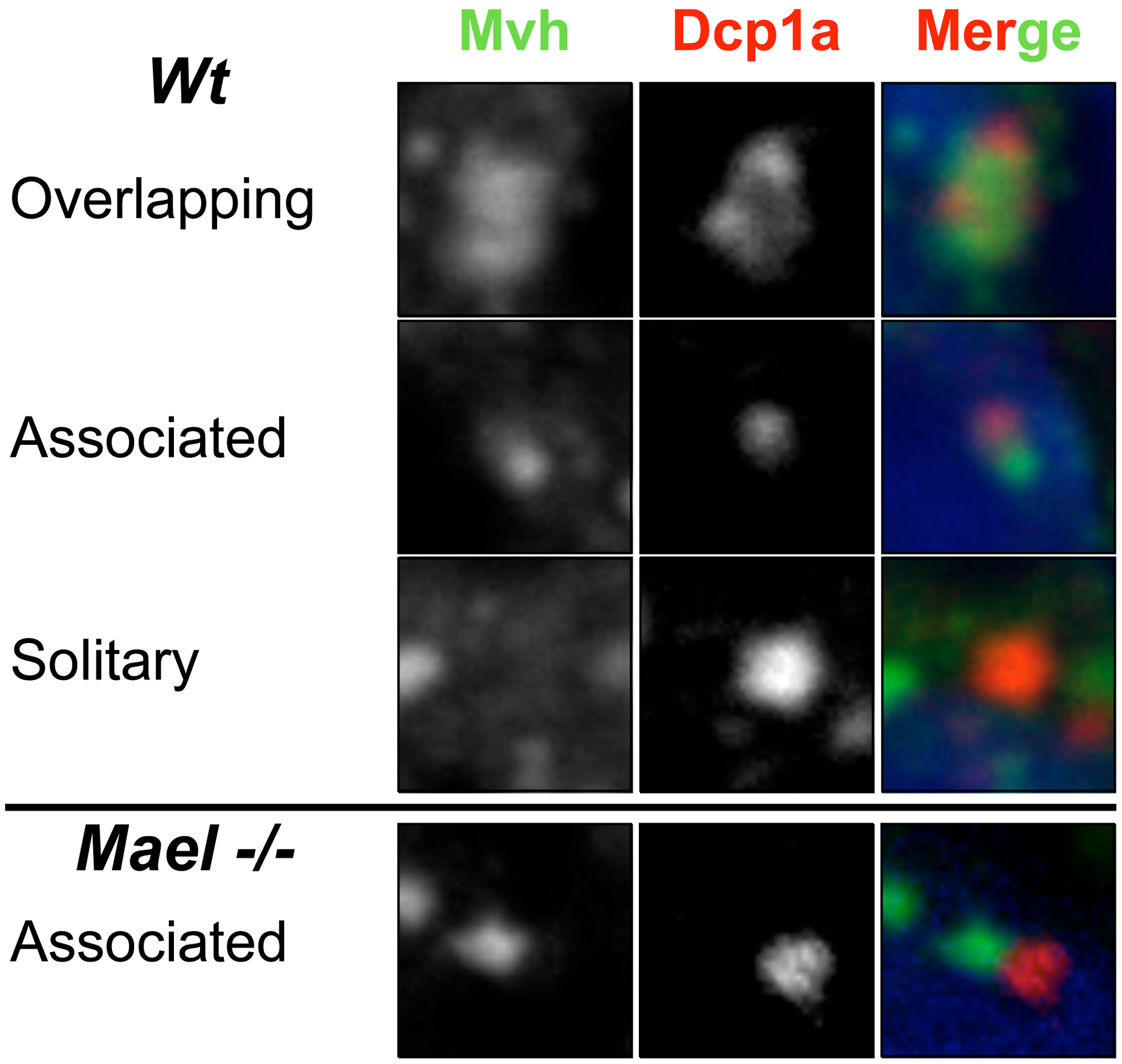

Supplement: Figure S5 — Associations of MVH and DCP1a in wild-type and Mael-mutant gonocytes. In wild-type gonocytes, 3 types of MVH-DCP1a localizations were observed: 1) overlapping, 2) associated granules, or 3) solitary granules. In the Mael mutant, virtually only associated or solitary DCP1a granules were present. (1.18 MB TIF) [file pgen.1000764.s005.tif]

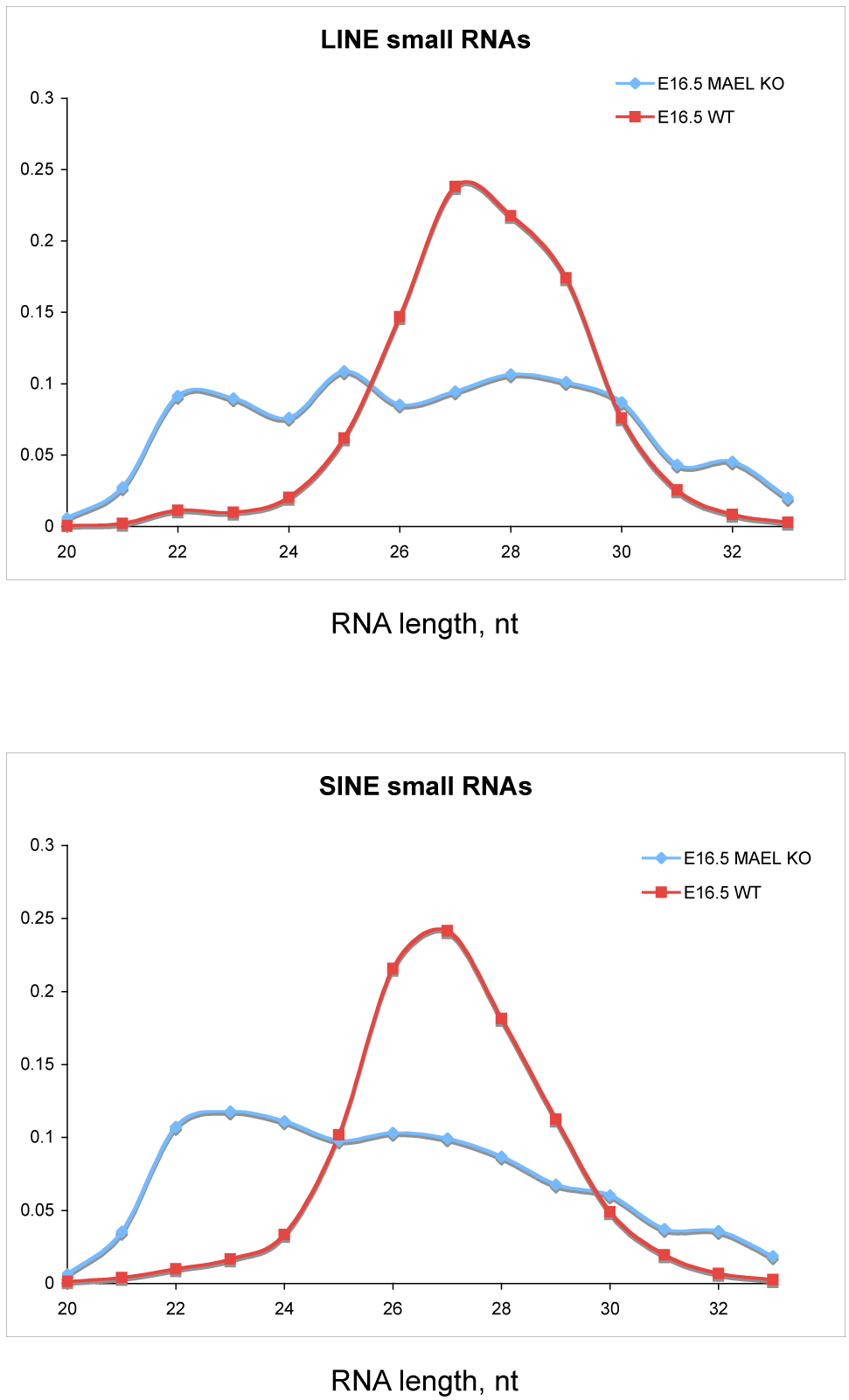

Supplement: Figure S6 — Size distributions of LINE and SINE small RNAs from E16.5 Mael-mutant testes. (0.46 MB TIF) [file pgen.1000764.s006.tif]

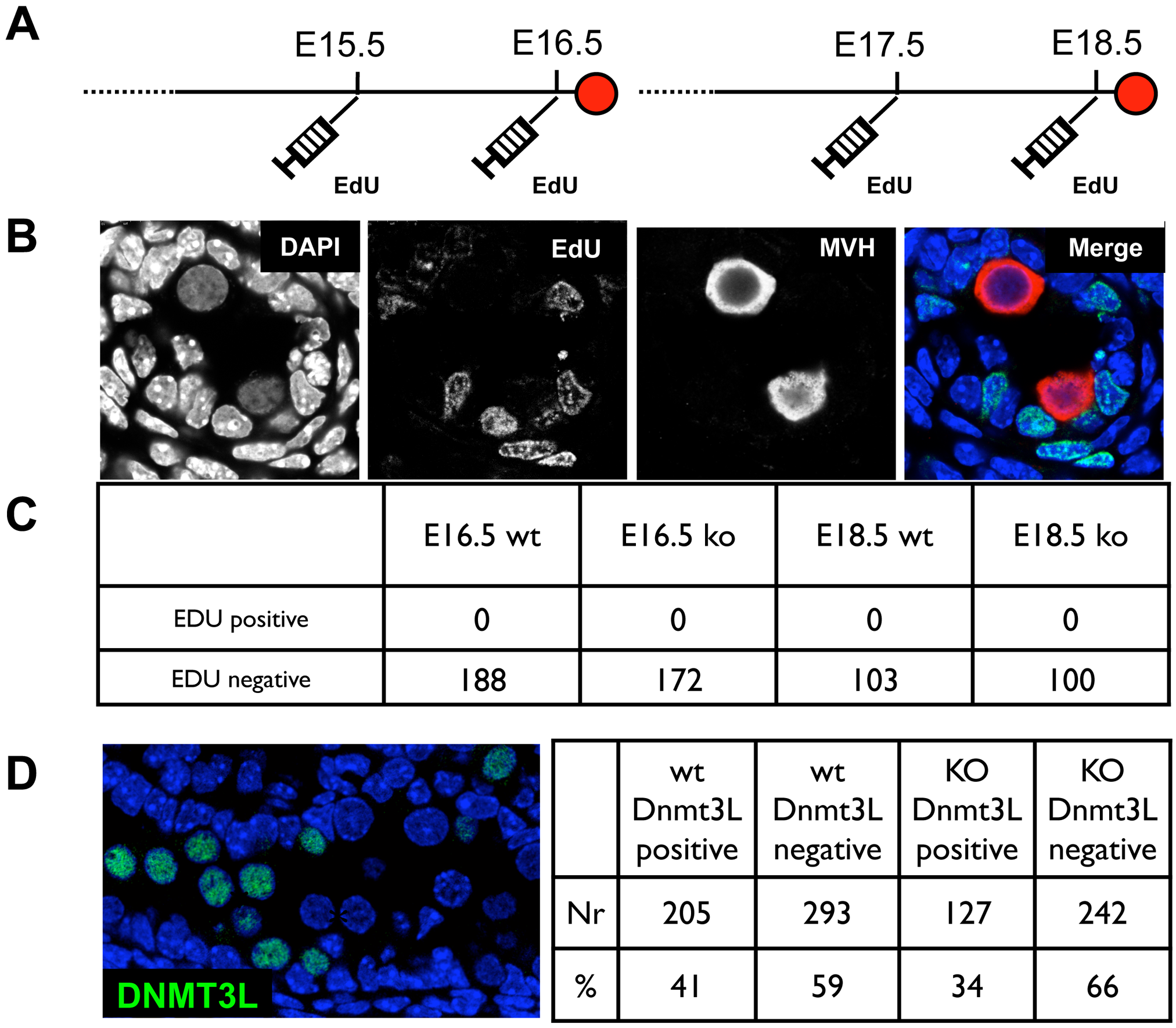

Supplement: Figure S7 — Analysis of gonocyte cell cycle arrest in wild-type and Mael-mutant animals by EdU labeling of replicating DNA. (A) Schematic outline of the experiment–timing of EdU injections in two groups of animals and their sacrifice. (B) Representative DAPI/EdU/MVH staining of processed gonadal tissues. No EdU labeling was observed in gonocytes. (C) Quantification of EdU labeling in gonocytes. No EdU positive wild-type or Mael-mutant gonocytes were observed. (1.56 MB TIF) [file pgen.1000764.s007.tif]

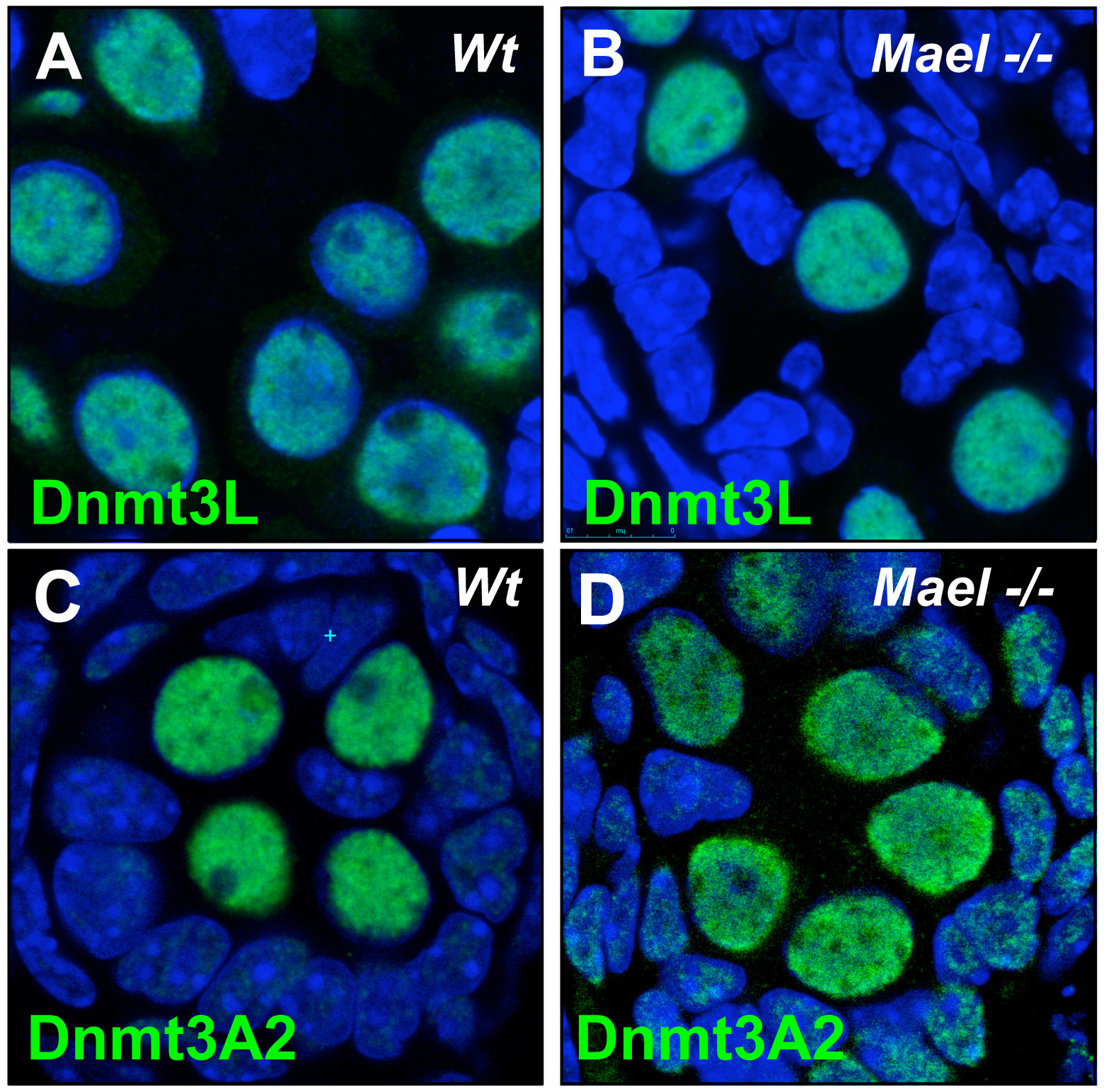

Supplement: Figure S8 — DNMT3L and DNMT3A2 expression in E16.5 Mael-mutant gonocytes. At day E13.5, no DNMT3L and DNMT3A2 staining was observed in wild-type and Mael-mutant gonocytes (data not shown). At day E16.5, all gonocytes showed prominent nuclear staining. (3.82 MB TIF) [file pgen.1000764.s008.tif]

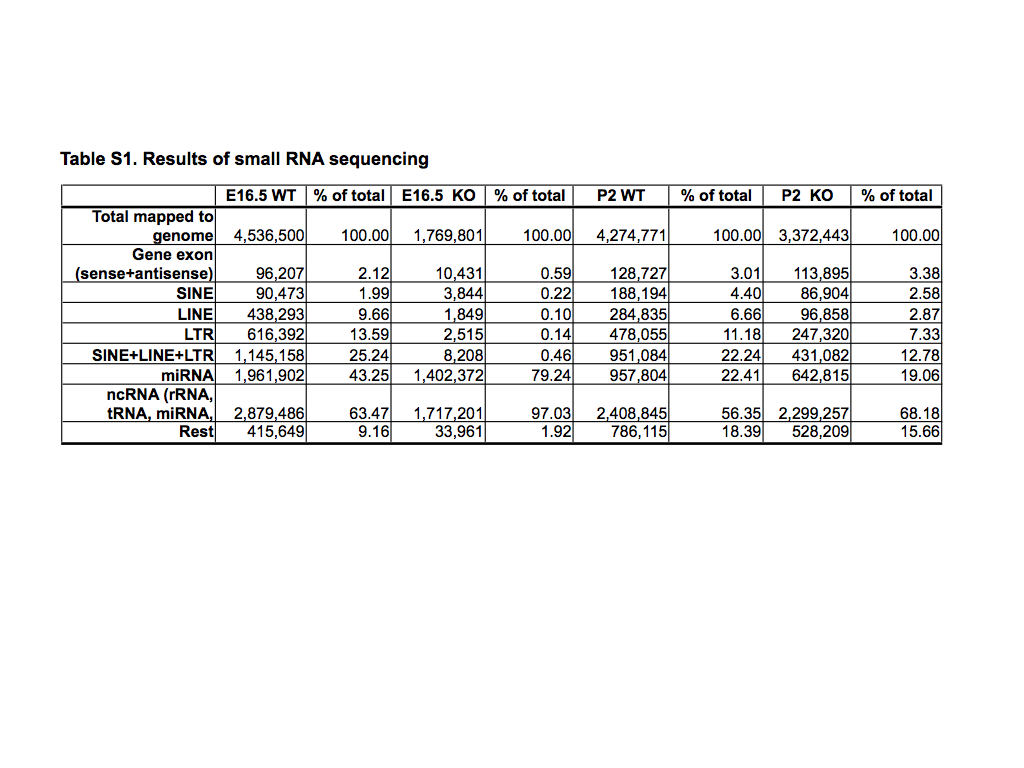

Supplement: Table S1 — Results of small RNA sequencing. (0.14 MB TIF) [file pgen.1000764.s009.tif]
